# Visualization of nanoscale magnetic domain states in the asteroid Ryugu

**DOI:** 10.1038/s41598-023-41242-x

**Published:** 2023-08-29

**Authors:** Yuki Kimura, Takeharu Kato, Toshiaki Tanigaki, Tetsuya Akashi, Hiroto Kasai, Satoshi Anada, Ryuji Yoshida, Kazuo Yamamoto, Tomoki Nakamura, Masahiko Sato, Kana Amano, Mizuha Kikuiri, Tomoyo Morita, Eiichi Kagawa, Toru Yada, Masahiro Nishimura, Aiko Nakato, Akiko Miyazaki, Kasumi Yogata, Masanao Abe, Tatsuaki Okada, Tomohiro Usui, Makoto Yoshikawa, Takanao Saiki, Satoshi Tanaka, Fuyuto Terui, Satoru Nakazawa, Hisayoshi Yurimoto, Takaaki Noguchi, Ryuji Okazaki, Hikaru Yabuta, Hiroshi Naraoka, Kanako Sakamoto, Sei-ichiro Watanabe, Yuichi Tsuda, Shogo Tachibana

**Affiliations:** 1 Institute of Low Temperature Science, Hokkaido University, Sapporo, 060-0819 Japan; 2 Nanostructures Research Laboratory, Japan Fine Ceramics Center, Nagoya, 456-8587 Japan; 3 Research and Development Group, Hitachi, Ltd., Hatoyama, Saitama 350-0395 Japan; 4 Department of Earth Sciences, Tohoku University, Sendai, 980-8578 Japan; 5 Department of Earth and Planetary Science, The University of Tokyo, Tokyo, 113-0033 Japan; 6 Institute of Space and Astronautical Science, Japan Aerospace Exploration Agency, Sagamihara, 252-5210 Japan; 7 National Institute of Polar Research, Tachikawa, 190-8518 Japan; 8 Kanagawa Institute of Technology, Atsugi, 243-0292 Japan; 9 Department of Natural History Sciences, Hokkaido University, Sapporo, 060-0810 Japan; 10 Division of Earth and Planetary Sciences, Kyoto University, Kyoto, 606-8502 Japan; 11 Department of Earth and Planetary Sciences, Kyushu University, Fukuoka, 819-0395 Japan; 12 Graduate School of Advanced Science and Engineering, Hiroshima University, Higashi-Hiroshima, 739-8526 Japan; 13 Department of Earth and Environmental Sciences, Nagoya University, Nagoya, 464-8601 Japan

**Keywords:** Planetary science, Early solar system, Meteoritics, Mineralogy, Astronomy and planetary science, Early solar system, Meteoritics, Mineralogy

## Abstract

In the samples collected from the asteroid Ryugu, magnetite displays natural remanent magnetization due to nebular magnetic field, whereas contemporaneously grown iron sulfide does not display stable remanent magnetization. To clarify this counterintuitive feature, we observed their nanoscale magnetic domain structures using electron holography and found that framboidal magnetites have an external magnetic field of 300 A m^−1^, similar to the bulk value, and its magnetic stability was enhanced by interactions with neighboring magnetites, permitting a disk magnetic field to be recorded. Micrometer-sized pyrrhotite showed a multidomain magnetic structure that was unable to retain natural remanent magnetization over a long time due to short relaxation time of magnetic-domain-wall movement, whereas submicron-sized sulfides formed a nonmagnetic phase. These results show that both magnetite and sulfide could have formed simultaneously during the aqueous alteration in the parent body of the asteroid Ryugu.

## Introduction

Rocks acquire remanent magnetization that reflects the environments that they have experienced, and they can retain this magnetization over the age of the Solar System^1^. Therefore, by studying the remanent magnetization of extraterrestrial materials such as meteorites, mass accretion rates at the time that the Solar System formed have been determined from the nebular magnetic field^2^, and the maximum temperatures experienced by various minerals have been estimated from the relaxation temperatures of their metastable magnetic domain (transdomain) structures^3^. In studies of remanent magnetization, the remanence vectors of entire bulk rock samples are measured by stepwise demagnetization, and the remanence carriers of demagnetization segments are interpreted on the basis of the macroscopic magnetic properties of the magnetic minerals. However, the link between macroscopic magnetic properties and tiny magnetic mineral particles is ambiguous, and direct observation of the morphology and magnetic-domain structure of magnetic minerals is clearly required to achieve a precise interpretation of the remanence record and to provide insight into the nebular environment at the time and place where each mineral was formed.

In the analyses of meteorites, depending on the period between the time that meteorite fell to the Earth and the time at which an analysis is performed, it becomes more difficult to identify the original natural remanent magnetization due to the viscous remanent magnetization caused by the Earth's magnetic field and mineral alterations caused by terrestrial weathering of the sample. Therefore, it is sometimes difficult to determine whether magnetic minerals have retained their original magnetic-domain structure or whether the main component of the remanent magnetization of the meteorite was acquired in the parent body.

In contrast, samples collected from the near-Earth C-type asteroid (162173) Ryugu by the Hayabusa2 expedition and brought back to the Earth in December 2020 have a clear history, and have only been exposed to the Earth's magnetic field and atmosphere for a short time. Details of the origin of the samples, such as where and how they were collected on the surface of Ryugu, are also known, and their temperature history (< 65 °C; lower than the daytime surface temperature of the asteroid) after the collection is clear^4,5^.

The samples from the asteroid Ryugu correspond to carbonaceous chondrites of the Ivuna (primitive meteorite) type^6–10^. It would be expected that magnetic minerals such as magnetite, sulfides, metallic iron, or iron–nickel alloys present in the carbonaceous chondrites would display natural remanent magnetization. In fact, systematic measurements of the bulk remanent magnetization of the Ryugu samples showed that the ferromagnetic minerals present are fine-grained magnetite particles with sizes in the submicron-to-micron range, pyrrhotite particles with sizes in the submicron to several-hundred micron range, and micron-sized or larger coarse-grained magnetite particles, in descending order of their contribution to the remanent magnetization^11^.

In the case of the Ryugu samples, the major carrier of remanent magnetization is reported to be magnetite formed in a nebular magnetic field environment of 41–390 μT^11^. Characterization of the magnetite has shown that the mass accretion that created the ~ 100 μT nebular magnetic field occurred at the time and place at which the magnetite was formed. This provides an endorsement of the discussion of the physical evolutionary processes of planetary systems discussed in bulk remanent-magnetization studies^12–21^. The paleomagnetic measurements for two Ryugu particles showed the presence of stable remanence components in the middle-coercivity range, and the carrier of stable components can be interpreted to be fine-grained framboidal magnetite; however, this interpretation remains uncertain. The origin and stability of a remanence record is critically dependent on the origin of the corresponding ferromagnetic mineral, its magnetic-domain structure, and the physical/chemical history of the natural sample. Thus, a knowledge of the mineralogical texture and magnetic-domain structure provides critical constraints for the interpretation of remanence records. We therefore used electron holography to observe the magnetic-domain structures of the magnetite, iron sulfide, and iron–nickel alloy that are the representative magnetic minerals in the Ryugu samples.

## Results

First, sample C0002-FC019, collected at the second touchdown site, was fixed on an indium metal substrate [Supplementary Fig. S1A] was observed using a tabletop scanning electron microscope (SEM) (JCM-7000, NeoScope, JEOL Ltd., Tokyo) equipped with an energy-dispersive X-ray spectrometer (EDS) (Supplementary Fig. S1B–D). The acceleration voltage for SEM–EDS was set as low as possible (5 kV) to minimize any influence on the magnetic field on the sample, and characteristic X-rays with energies above 3.5 kV were detected. We analyzed the samples under weak-magnetic-field conditions comparable to or lower than the Earth's magnetic field (about 50 μT) until the electron holography observation was completed. The bulk elemental composition of C0002-FC019 obtained under these conditions was O:Na:Mg:Al:Si:S:Fe:Ni = 53:1:13:1:16:4:9:1 (Supplementary Table S1). These are raw data values that have not been corrected by using a correction factor. Here, C was excluded from the measurements, because the sample was coated with carbon to prevent electrical charging during the SEM observations. Also, K and Ca were not detected due to the low acceleration voltage. The positions of magnetite and iron sulfide, which are typical magnetic minerals, were searched in the SEM and EDS mapping images (Supplementary Fig. S1). It was difficult to distinguish and identify individual mineral species on the micrometer scale by low-voltage analysis with the tabletop SEM instrument. Therefore, to prepare transmission electron microscope (TEM) samples for electron holography, we selected a region of dense spherical particles about 1 μm in diameter that contained iron and oxygen, as these particles were the most likely to consist of framboidal magnetite. Similarly, regions containing iron and sulfur and a high nickel content were selected as particles with a high possibility of consisting of iron sulfide. Altogether, a total of nine regions were selected (Supplementary Table S1 and Supplementary Fig. S1).

The sample C0002-FC019 measured 160 × 130 μm in size and had at least 281 μm^2^ of iron-enriched area out of the 1.44 × 10^4^ μm^2^ of surface available for analysis. Assuming that this was all magnetite, magnetite occupied at least 1.95% of the surface. This is comparable to the value of 3.7 ± 1.7 vol.% for the nine samples in the sample catcher-C, analyzed in the initial curation analysis^22^.

### Magnetite

**Figure 1:**
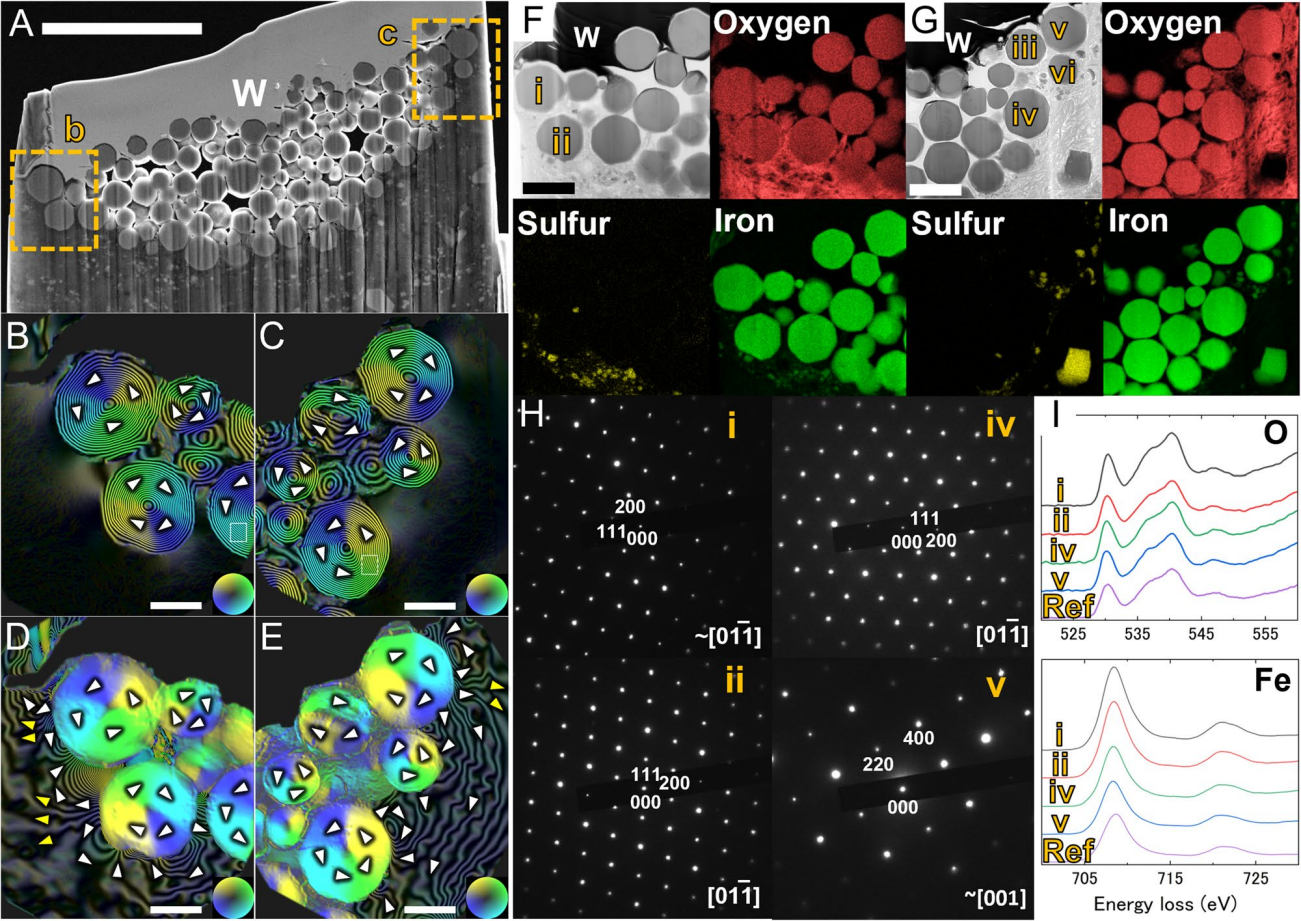
Magnetic structure of framboidal magnetite particles. (**A**) Secondary electron image of a thin section extracted from region i in SI Fig. S1. This image was observed after the electron holography observations shown in (**B**–**E**). The region W with a uniform contrast is a deposited layer of tungsten to prevent beam damage during FIB machining. Scale bar: 5 μm. (**B**,**C**) Color-contour maps of the remanent magnetic states of magnetites corresponding to rectangles b and c in *A*, respectively. There was a constant flux of *h*/*e* Wb flow between adjacent contour lines, where *h* is the Planck constant and *e* is the elementary charge. Each particle had a concentric circular magnetic field and a closed (i.e., vortex) structure. Only a magnetite shown by a yellow arrow in *C* has a tilted vortex state from the parallel to the electron beam. (**D**,**E**) Twenty-times phase-amplified reconstructed images of (**B**) and (**C**), respectively. Scale bars: 500 nm. The corresponding magnetic and electrostatic phases are shown in SI Fig. S2. (**F**,**G**). STEM bright-field images near the regions b and c, respectively, after further thinning and corresponding elemental mapping. Further images are given in SI Fig. S3. Scale bars: 1 μm. (**H**) Electron-diffraction patterns corresponding to i, ii, iv, and v in (**F**) and (**G**). The brackets indicate the crystal zone axes. The sample was slightly tilted to orient the crystal zone axis parallel to the electron beam. Appropriate indices appear just below each spot. (**I**) EELS spectra for oxygen (upper) and iron (lower) corresponding to particles i, ii, iv, and v in (**F**) and (**G**). The bottom spectra, labeled ‘Ref’ in each case, are taken from a synthesized magnetite (99.5% in purity; Kojundo Chemical Lab. Co., Ltd.). The atomic ratios of oxygen and iron for each magnetite particle are listed in SI Table S2.

To minimize the loss of the original magnetic state in the magnetite particles, a relatively thick thin-section of ~ 400 nm was extracted from region i (Supplementary Fig. S1C) by using a focused ion beam (FIB)-SEM system (NB5000; Hitachi High-Tech Corp., Tokyo). Several attempts to observe relatively thick samples with electron holography at high acceleration voltages have been made effectively^23,24^. The sample preparation was performed using a cryo stage (− 90 °C) to prevent FIB damage to the matrix surrounding the magnetites and without SEM mode to avoid the addition of artificial magnetic components. This thin section contained a framboid, composed of numerous magnetite particles each with a diameter of ~ 1 μm, within a dimple ~ 15 μm in diameter and ~ 5.5 μm in depth (Fig. 1A). At least 134 magnetite particles were present in the thin section, the largest of these being ~ 1.3 μm in diameter; typical sizes were 0.6–0.9 μm. The SEM image reproduced in Supplementary Fig. S1D showed that the corresponding region had the texture of a typical framboid. If the dimple is assumed to form part of a sphere, its estimated total volume is ~ 600 μm^3^. The framboids are then estimated to have consisted of a total of ~ 3 × 10^3^ magnetite particles. This is comparable to typical framboidal magnetites found in meteorites^25^. We performed measurements on 712 magnetite particles in the Ryugu samples (A0064, C0106) observed by our initial analysis by conventional SEM; these particles had diameters ranging from 96 to 1505 nm and an average grain diameter of 637 ± 247 nm (1σ). Therefore, the magnetite particles shown in Fig. 1 are typical of framboidal magnetites in the Ryugu sample.

Magnetite particles around both the ends of this thin section (the square regions in Fig. 1A) were examined by electron holography using a TEM equipped with a Lorentz stage installed at Hitachi Ltd. in Hatoyama, Japan. The operational acceleration voltage was 1 MV. The observed magnetite particles showed a characteristic vortex structure (Fig. 1B,C and Supplementary Fig. S2), indicating that some of the magnetic flux had leaked out of the particles (Fig. 1B–E). This is similar to the six magnetites of A0064 observed in the initial analysis^7,26^. The vortex-like magnetic-domain structure of this magnetite had both clockwise and counterclockwise magnetic domains in similar ratios. Moreover, neighboring grains tended to show opposite vortex domains to reduce stray field energy among the grains. Note that since most of the magnetite is larger than 400 nm in diameter, some of it has been truncated, as suggested by the flat contrasts in the electrostatic phase of the central part of the particles (Supplementary Fig. S2). Thinning of magnetite may have affected the original magnetic domain structure, since the vortex structure of magnetite except for a particle indicated by an arrow in Fig. 1C is normal to the sample plane. It should be noted, however, that all unprocessed magnetites in the Tagish Lake meteorite also had similar vortex states parallel to the electron beam^27^.

After the electron holography observation, the thin section was thinned further to ~ 70 nm around both the ends at −90 °C for basic analysis by conventional TEM with an acceleration voltage of 200 kV, in which a strong magnetic field was applied to the sample (Supplementary Fig. S3A). Scanning TEM (STEM)-EDS elemental mapping and electron-diffraction patterns showed that the spherical particles consisted of single crystals of magnetite (Fig. 1F–H, and Supplementary Fig. S3B, S3C, and S4). In the previous study, all the magnetite was found to contain nickel [Ni/(Fe + Ni) =  ~ 0.05]^22^, whereas in this study, the amount of Ni was below the detection limit [Ni/(Fe + Ni) < 10^−3^]. It is possible that the SEM–EDS analysis in the previous study detected Ni-derived signals in the matrix region around magnetite. SEM observations of our bulk sample also showed Ni contents of 0.01–0.03 in regions i, vi, and viii, which are thought to consist of magnetite. However, from the STEM analysis with high spatial resolution, we conclude that the framboidal magnetite contained almost no Ni. This is consistent with the sharp indication of a Verwey transition at around 120 K in the low-temperature remanence curves of the bulk magnetic measurements^11^, which indicated that stoichiometric magnetite was present^28^. Electron energy-loss spectroscopy (EELS) analysis of the electronic states of iron and oxygen also showed typical spectra of magnetite, with no evidence of oxidation (Fig. 1I), which was also consistent with the low-temperature remanence curves of the bulk measurements^11,29^. The matrix surrounding magnetite was a layered silicate composed mainly of silicon and magnesium with a Mg:Fe:Si:Al ratio of 47.0:6.1:44.0:2.9 in the square region of Supplementary Fig. S4B; this is close to the ratio for saponite, which contains interlayer H_2_O in addition to structural OH sites.

### Pyrrhotite

Iron sulfide can adopt various crystal structures depending on its composition ratio. In addition to pyrrhotite (formed by aqueous alteration), a small amount of pentlandite was found in the Ryugu samples, and the abundance of iron sulfide has been reported to be 3.3 − 5.1 vol.% for C0002^7^. Pyrrhotite (Fe_1-x_S) has a magnetic susceptibility that varies with the ratio of Fe to S. In particular, Fe_7_S_8_ (4C), Fe_9_S_10_ (5C), and Fe_11_S_12_ (6C), known for 0.08 ≤ x ≤ 0.125, exhibit ferrimagnetism and have the potential to acquire remanent magnetization. On the other hand, in the case 0 ≦ x ≦ 0.08, pyrrhotite becomes antiferromagnetic and does not contribute to remanent magnetization.

**Figure 2:**
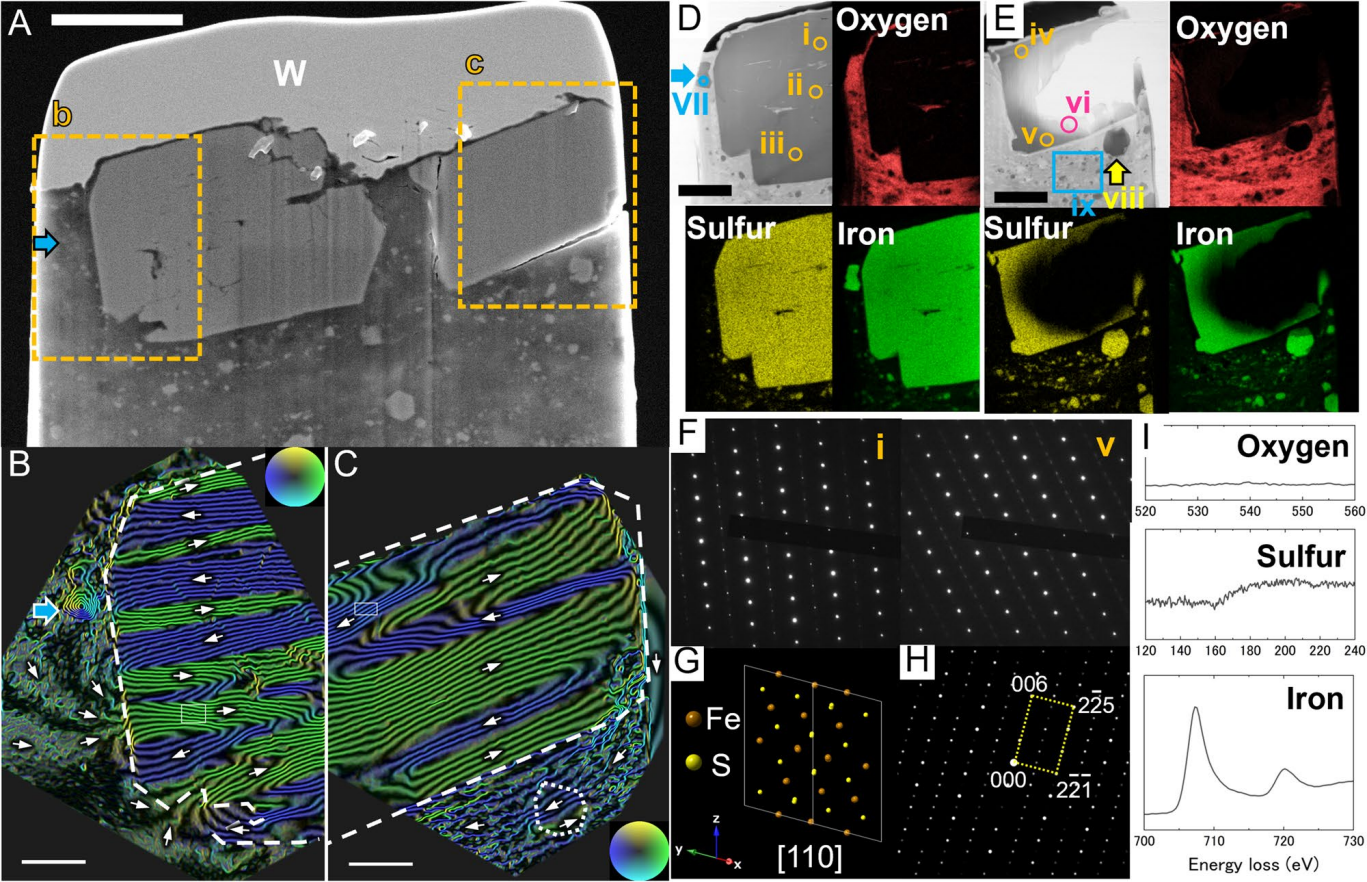
Magnetic structures of pyrrhotite. (**A**) Secondary electron image of a thin section extracted from region ii in SI Fig. S1. The arrow shows a relatively smaller magnetite particle in the matrix. Scale bar: 2 μm. (**B**,**C**) Color-contour maps (amplified four times) of the remanent magnetic states of pyrrhotites in rectangles b and c in (**A**), respectively, with a constant flux of *h*/4*e* Wb flow between adjacent contour lines. Each particle had a multidomain structure. The dashed lines indicate the outline of the pyrrhotite particles. The magnetite particle shown by the cyan arrow in *B* had a vortex magnetic structure. The dotted line in C shows the position of the iron sulfide particle. Scale bars: 500 nm. Corresponding magnetic and electrostatic phases are shown in SI Fig. S5. (**D**,**E**) STEM bright-field images for the regions around *b* and *c*, respectively, after further thinning and corresponding elemental mapping. The overall image is shown in SI Fig. S6. Scale bars: 1 μm. (**F**) Electron diffraction patterns corresponding to i and v in *D* and *E*. The sample was tilted to orient the crystal zone axis parallel to the electron beam. The tilt angles (TX, TY) were (18.7, 16.6) for i and (20.3, −17.5) for v. All the diffraction patterns are given in SI, Fig. S7. (**G**) Computational projection of atoms for polytype-4C, Fe_7_S_8_, of pyrrhotite in view [110]. (**H**) Simulated diffraction pattern corresponds to *G*, which is consistent with the diffraction patterns in (**F**). (**I**) EELS spectra of oxygen, sulfur, and iron from top to bottom, respectively, obtained from the circle vi in (**E**). Elemental ratios corresponding to i–v and vii are given in SI Table S3.

A 400-nm-thick section, extracted from region ii in SI, Fig. S1C, that was expected to consist of iron sulfide based on the SEM observations, is shown in Fig. 2A. There are two samples with sizes of 4.3 μm × 3.1 μm and 2.9 μm × 2.1 μm, surrounded by faceted surfaces. The near-vacuum regions of these two particles were observed by 1 MV holography TEM (Fig. 2B,C and Supplementary Fig. S5). Both particles were found to have 180-degree multidomain structures. The particles in Fig. 2B,C consisted of at least 12 and 6 magnetic domains, respectively, and the analysis reported below showed that each magnetic flux was parallel to the c-plane of the pyrrhotite crystal for both crystals (see next paragraph). Regions b and c were both pyrrhotite crystals with the same crystal structures, whereas their crystallographic orientation and magnetic flux directions were slightly different. This suggests that the external magnetic field was not strong enough to affect the crystalline orientation of the growing pyrrhotite crystals. Bulk magnetic measurements showed that the pyrrhotite does not carry a stable remanent magnetization^11^.

Although the theoretical estimation of multidomain magnetite predicted the highly stable remanence in case of small particle size and large Barkhausen volume^30^, larger size of the pyrrhotite and its low-Curie temperature likely reduce the remanence stability of multidomain state. Therefore, the timescale of magnetic-domain-wall movement of multidomain pyrrhotite to change the remanence is likely much shorter than 4.6 billion years, as remanent magnetization is lost over a period of 4.6 billion years.

After the electron holography observations, the thin section was cooled to −90 °C and further thinned by using the FIB to produce ultrathin sections for further analysis (Supplementary Figs. S6A and S7A). Elemental mapping by STEM-EDS indicates that the two particles both consisted of iron sulfide (Fig. 2D,E; Supplementary Figs, S6B and S6C). Electron-diffraction patterns were also obtained (Fig. 2F and Supplementary Fig. S7B) and compared with diffraction patterns simulated by using CrystalMaker X and SingleCrystal 4 (Fig. 2G,H); the patterns were found to correspond to Fe_7_S_8_ (4C) with a monoclinic crystalline structure, as expected [JCPDF 29-723]. Fe_7_S_8_ (4C) is a typical phase of relatively large iron sulfide particles in the Ryugu sample^7^. EELS analysis of the spectra corresponding to the energy loss in region vi of the iron sulfide in Fig. 2E, which depends on the electronic states of O, S, and Fe (Fig. 2I), showed no signal due to O, indicating that the iron sulfide was pure and was not oxidized.

### Fe–Ni sulfide

**Figure 3:**
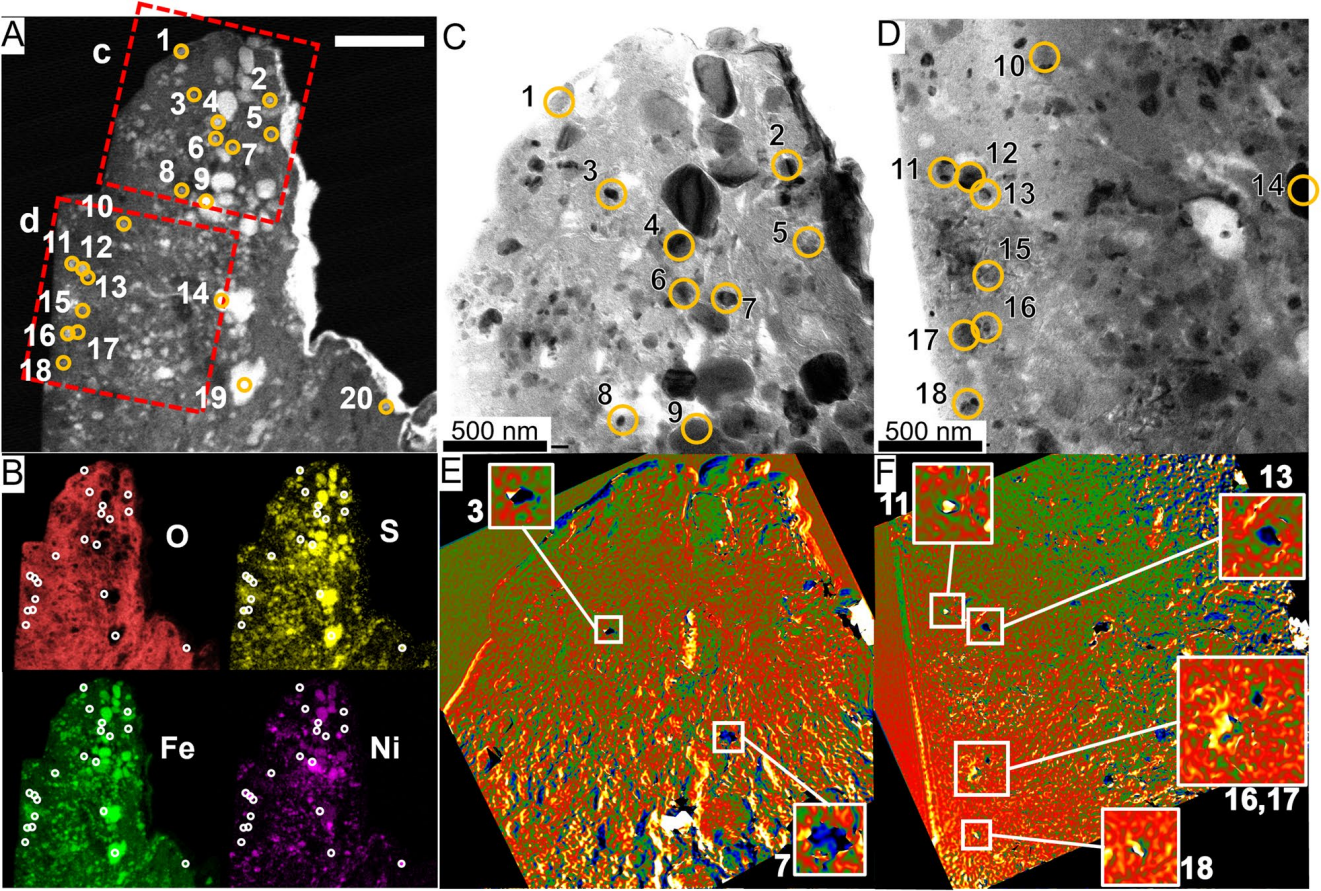
A thin section of a nickel-rich region. (**A**) HAADF image of a thin section extracted from region iii in SI Fig. S1. Tiny particles were distributed in the matrix. Scale bar: 1 μm. (**B**) Corresponding elemental mappings observed by STEM–EDS. The elemental ratios corresponding to circles 1–18 are listed in SI Table S4. (**C**,**D**) TEM images of the region indicated by squares c and d in (**A**). (**E**,**F**) Colored phase images (8 times the phase-amplified reconstruction) showing the magnetic structures corresponding to (**C**) and (**D**), respectively. There were no characteristic strong magnetic structures that had a unique direction. The tiny particles indicated by squares and in the enlarged images had a concentric circular magnetic field and a closed (i.e., vortex) structure. Further images are given in SI Figs. S8 and S9.

A magnified STEM high-angle annular dark-field (HAADF) image of a 150 nm-thick ultrathin section (Supplementary Fig. S8) extracted from the Fe- and Ni- rich region iii (Supplementary Fig. S1C) is shown in Fig. 3A. EDS mapping revealed the presence of numerous microscopic Fe sulfide particles, as well as Fe–Ni sulfides (Fig. 3B and Supplementary Fig. S9). The Fe–Ni ratios [Ni/(Fe + Ni)] of pyrrhotite and pentlandite [(Fe,Ni)_9_S_8_], determined by SEM–EDS analysis and reported in a previous study were 0–0.14 and 0.20–0.57, respectively. The Fe–Ni ratio in region iii observed by SEM was 0.12 (Supplementary Table S1). The Fe–Ni ratios of individual particles, determined by STEM-EDS analyses, were distributed in the range 0.01–0.45 (Supplementary Table S4). Figure 3C,D are enlarged TEM images of squares c and d, respectively, in Fig. 3A. Many crystalline particles with strong contrasts of less than 300 nm were observed. Most of these particles consisted of Fe–Ni sulfide. Colored phase images obtained by electron holography of this region showed no significant magnetic flux or magnetic-domain structure (Fig. 3E,F). On the other hand, vortex magnetic domains were observed in the area surrounded by squares in Fig. 3E,F. We can summarize our results by stating that most of the sulfide particles distributed in the matrix that do not have an external magnetic field and a magnetic-domain structure are not ferromagnetic phases and do not contribute to the remanent magnetization.

## Discussion

By examining the phase reconstructed from holograms in regions B and C with a 20-fold amplification, we found that the fluxes are *h*/4*e* Wb toward the left-hand side and *h*/10*e* Wb toward the right-hand side of the images, respectively (Fig. 1). The sum of these two values indicates that the framboidal magnetite has a flux of 3* h*/20*e* Wb toward the left-hand side of the image, corresponding to ~ 300 A m^−1^. The flux closure is not complete in local regions within the framboids, and the framboid likely carries a net remanence of Ryugu particle. The bulk remanent magnetization of C0002-4-f, which originated from the same mother particle as our sample, has been measured to be 0.02 A m^2^ kg^−1^ (11). By a measured density of 1790 kg m^−3^ for C0002, the strength of magnetization is calculated to be 35.8 A m^−1^. The dispersion of magnetization directions amongst the framboids would reduce the bulk magnetization by a factor of 10 relative to the localized estimate. The degree of remanence reduction, i.e. dispersion of magnetization directions, should relate to the remanence acquisition mechanism and the intensity of external field, and the roughly estimated factor 10 reduction would give an important constraint on the future study investigating the remanence acquisition mechanism of carbonaceous chondrites.

The stable component of natural remanent magnetization in the medium-coercivity range is interpreted as being carried by framboidal magnetite particles of submicron-to-micron size^11^. Our analysis clearly indicates that the framboidal magnetite particles have a vortex magnetic-domain structure, probably with alternating vortex directions. The framboidal magnetite particles with diameters ranging from 96 to 1505 nm in the Ryugu particle that crystallized and increased in grain size during aqueous alteration showed a single-vortex state, which is consistent with the numerical calculation that the single-vortex state is confirmed to persist until ~ 1500 nm for increasing grain size in equidimensional spheres and cuboctahedra magnetites^31^. The numerical model of thermal activation showed that equidimensional cuboctahedral magnetite particles with grain sizes in excess of ~ 100 nm have a single-vortex state and can retain stable remanence over billions of years^32^. Thus, the framboidal magnetites with a single-vortex state in the Ryugu particles could have retained a stable remanence during 4.6 Gyr, which is consistent with the interpretation that the framboidal magnetites retained a remanence of the nebular field^7,11^. In terms of remanence stability, if the vortex direction of neighboring magnetite grains is fixed due to the magnetostatic interaction, the energy barriers between two states with different magnetization directions might increase and, consequently, the remanence stability of the magnetite grains in framboid might be greater than that of isolated magnetite grains.

Magnetite may form by homogeneous nucleation from solution^27^, by precipitation after dissolution of iron sulfide, or by oxidation of metallic iron^33^. Iron sulfide is present just outside the magnetite particles in Fig. 1F,G. In particular, both pure magnetite and iron sulfide exist close to one another (~ 1 μm) in the region shown in the lower right-hand side of Fig. 1G. This might be the result of the formation of magnetite particles through nucleation and precipitation from solution after the matrix underwent aqueous alteration to form phyllosilicates, rather than through dissolution and precipitation of iron sulfide or by metal oxidation. The vortex magnetic-domain structure with an alternating vortex direction can be explained by at least two different formation mechanisms. First one is that the magnetite grains formed a framboidal texture before the individual grains grew larger than the blocking volume (~ 100 nm)^32^. The second one is that a framboid is formed from magnetite particles that are already in a vortex state and self-assembled by particle–particle interactions as a framboid is assembled^27^. Both mechanisms support the hypothesis that the framboidal magnetite formed through nucleation and precipitation from solution after the matrix underwent aqueous alteration. In this scenario, the magnetite grains below the blocking volume with a framboidal texture grew and acquired remanent magnetization at the critical-grain size.

The pyrrhotite crystals in the region b in Fig. 2A contained inclusions of magnesium silicate on the c-plane (Fig. 2D and Supplementary Fig. S6B). The two main locations of the inclusion are between i and ii and between ii and iii, where electron-diffraction patterns were obtained. The slight deviation in the crystallographic axes of i–iii (Supplementary Fig. S7) suggests that the inclusion was not introduced after pyrrhotite formation, but rather during its formation. The pyrrhotite grew faster than the magnesium silicate dissolved, i.e., it formed at a larger growth rate. A similar size of fluid inclusion was found in a previous study^7^, suggesting a similarly large growth rate for pyrrhotite formation. As it is not easy to incorporate large inclusions during the growth of a single crystal, we can speculate that the iron sulfide formed within a timescale similar to that of laboratory experiments. Phyllosilicates could be seen extending into the inner matrix region beyond the iron sulfide (Supplementary Fig. S6). The coexistence of iron sulfide and layered silicates indicates that both formed simultaneously.

Bulk magnetic measurements have shown that the pyrrhotite does not have a stable remanence component^11^. Our observations indicate that the pyrrhotite with a large grain size had a multidomain magnetic structure and most of the iron sulfide particles with small grain sizes formed a nonmagnetic phase. Therefore, the pyrrhotite with a high coercivity detected in the bulk magnetic measurements was probably multidomain pyrrhotite, and relaxation during 4.6 billion years might account for the failure to detect any natural remanent magnetization component in the pyrrhotite. These results indicate that the reason that magnetite displays natural remanent magnetization whereas pyrrhotite does not do so, is not because pyrrhotite formed at a different time from magnetite, when the nebular magnetic field in the environment where the Ryugu parent body was located was absent or weak, but rather that it could have been formed at the same time but has subsequently lost its remanent magnetization.

## Methods

The Hayabusa2 spacecraft collected samples from two locations on the surface of the asteroid Ryugu^4,5,34^. The first sample was collected when the spacecraft landed directly on the original surface; this sample was stored in chamber-A of the sample catcher. The second sample was collected when the spacecraft landed 20 m north of an artificial crater formed by shooting a small impactor from the spacecraft^35^; this sample was stored in chamber-C of the sample catcher. The sample catcher was fabricated from a nonmagnetic aluminum alloy^36^. The larger particles recovered from chamber-A and -C were labeled with the initials A and C, respectively, together with a four-digit number, and each piece was given a branch number. In this study, SEM observations were performed on 53 particles with lengths of 10 to 160 μm along their major axes {5 particles from A0063, 5 particles from A0064 [one of which was lost^26^], 3 particles from C0002, 5 particles from C0023, 32 small particles from C0040, and 3 particles from C0103}. Among these, we used particles C0002–FC019, which contained a variety of magnetic minerals on their surfaces and were large enough to permit the extraction of several ultrathin sections by FIB. C0002 was the third largest particle in the returned sample and this was also used for an initial analysis^6, 7^. The magnetic properties of C0002 were concluded to be representative of the surface material of the asteroid Ryugu^11^. FC019 is a fragment that was separated before C0002 was shared among the initial analysis team.

The Ryugu sample was mounted by placing it on an indium plate using an ultrafine brush and pressing it with a glass plate inside a glove box at Tohoku University (Supplementary Fig. S1A). The indium plate was attached by double-sided copper adhesive tape to a silicon plate no larger than 3 × 3 mm^2^, so that it could be placed on the specimen holder with a rotating base used in the FIB machining. The mounted sample was placed in a container filled with N_2_ gas; for analysis, this container was then placed in a purge-type glove box with a gas-recycle purification system (DBO-1NKPJO; Miwa Manufacturing Co., Ltd., Osaka) at the Japan Fine Ceramics Center. The glove box was capable of maintaining a dew-point temperature of approximately − 80 °C in Ar gas. All samples were kept in the glove box during the analysis period.

First, to suppress electrical charging during tabletop SEM observation, the sample on the indium plate was coated with carbon to a thickness of 30 nm by using a carbon coater (PECS II; Gatan, Inc., Pleasanton, CA). After the SEM observation, the sample was loaded into the FIB-SEM system and W was deposited on the selected regions i to ix in Supplementary Fig. S1C to protect the surface. Regions i, vi, and viii were most likely magnetite, regions ii and v were most likely pyrrhotite, and regions iii and ix had high nickel contents. After careful considerations based on, for example, considering ease of processing, three ultrathin sections were prepared from regions i–iii by using Ga + ions without the SEM mode of FIB-SEM system to prevent the presence of a magnetic field. Regions iv and vii were avoided because of their relatively high contents of magnesium and silicon. Each thin section prepared from the three selected regions was mounted on a Mo TEM grid, and the mounted specimens were cooled to − 90 °C for the preparation of ultrathin sections. Then, to minimize the effect of charging during electron holography observations, both sides of these ultrathin sections were coated with carbon to a thickness of 30 nm.

Among the three thin sections, thin sections i and ii, containing magnetite and pyrrhotite, respectively, were thickened to 400 nm to preserve the original magnetic-domain structure, and these were examined by using an ultrahigh-voltage TEM (H-1000FT; Hitachi, Ltd., Tokyo) in Hatoyama, dedicated to electron holography. The acceleration voltage was 1.0 MV. Several attempts to observe relatively thick samples with electron holography at high acceleration voltages have been made effectively. For example, magnetic flux and three-dimensional structure of skyrmion lattices in a 500-nm-thick sample and magnetic domain structures of a magnetic recording head in a 250-nm-thick sample were observed by electron holography at an acceleration voltage of 1 MV^23,24^.The samples were stored under Ar gas during their transport to Hitachi, Ltd. Ultrathin section iii, containing many microcrystals, was examined by holography TEM (HF-3300EH; Hitachi High-Tech Corp., Tokyo) with an acceleration voltage of 300 kV, located at the Japan Fine Ceramics Center.

In the observation using a 1 MV (300 kV) holography electron microscope, 10 (5) holograms were taken in each observation area with an exposure time of 30 (20) s. The reconstructed image of the electron wave passing through the sample contains information on the magnetic flux and the internal potential of the sample. To subtract the internal potential, the sample was turned over and a series of holograms were recorded again from the opposite side of the thin section. By averaging the 10 (selecting one image from each of five) images from the front and back sides and by subtracting the internal potential, we were able to visualize the nanometer-scale magnetic-domain structures and the flux distribution of magnetic minerals with a spatial resolution of 23 (14.4) nm. For more information, see Kimura, et al.^27^. Note that the averaging has been processed by averaging complex data of reconstructed object waves from the holograms using following equation^37^,

$$\frac{1}{J}{\sum }_{j}{a}_{j}{e}^{i({\varphi }_{j}+{\varphi }_{0j})}\to \langle a\rangle {e}^{i(\langle \varphi \rangle +{\widetilde{\varphi }}_{0})}$$

where *a*_*j*_, ${\varphi }_{j}$, and ${\varphi }_{0j}$ are amplitude, phase, and phase offset value of the reconstructed object wave from *j*th hologram. *J* is the total number of the holograms. Sample drifts in the images have been corrected and the phase offset values were adjusted to be same value ${\widetilde{\varphi }}_{0}$ before the averaging. The averaged phase $\langle \varphi \rangle$ was calculated from the averaged complex data $\langle a\rangle {e}^{i(\langle \varphi \rangle +{\widetilde{\varphi }}_{0})}$ and the phase jumps of 2π in the averaged phase image has been unwrapped. The merit of this method is the phase-unwrapping can be performed successfully comparing to do it for each reconstructed phase data from the hologram.

After we had examined the magnetic-domain structure by holography TEM, we performed STEM-EDS elemental mapping, electron diffraction, and EELS analyses by using conventional TEMs. All the sample-preparation and analysis stages were performed in an atmosphere-free environment, except for a total of less than 15 s of exposure to the atmosphere during sample loading and unloading to and from the tabletop SEM.

### Supplementary Information

Supplementary Information.

## Data Availability

All data needed to evaluate the conclusions in the paper are present in the paper and/or the Supplementary Materials.
