# Supplementary material for: Visualization of nanoscale magnetic domain states in the asteroid Ryugu
Source: Sci Rep. 2023 Aug 29;13:14096. doi: 10.1038/s41598-023-41242-x (PMC10465612; doi:10.1038/s41598-023-41242-x)
Supplement: Supplementary file 1 — Supplementary Information. [file 41598_2023_41242_MOESM1_ESM.docx]

**Supporting Information for**

**Visualization of nanoscale magnetic domain states in the asteroid Ryugu**

Yuki Kimura*, Takeharu Kato, Toshiaki Tanigaki, Tetsuya Akashi, Hiroto Kasai, Satoshi Anada, Ryuji Yoshida, Kazuo Yamamoto, Tomoki Nakamura, Masahiko Sato, Kana Amano, Mizuha Kikuiri, Tomoyo Morita, Eiichi Kagawa, Toru Yada, Masahiro Nishimura, Aiko Nakato, Akiko Miyazaki, Kasumi Yogata, Masanao Abe, Tatsuaki Okada, Tomohiro Usui, Makoto Yoshikawa, Takanao Saiki, Satoshi Tanaka, Fuyuto Terui, Satoru Nakazawa, Hisayoshi Yurimoto, Takaaki Noguchi, Ryuji Okazaki, Hikaru Yabuta, Hiroshi Naraoka, Kanako Sakamoto, Sei-ichiro Watanabe, Yuichi Tsuda, and Shogo Tachibana

*Yuki Kimura

Email: ykimura@lowtem.hokudai.ac.jp

This PDF file includes:

Figures S1 to S9

Tables S1 to S4


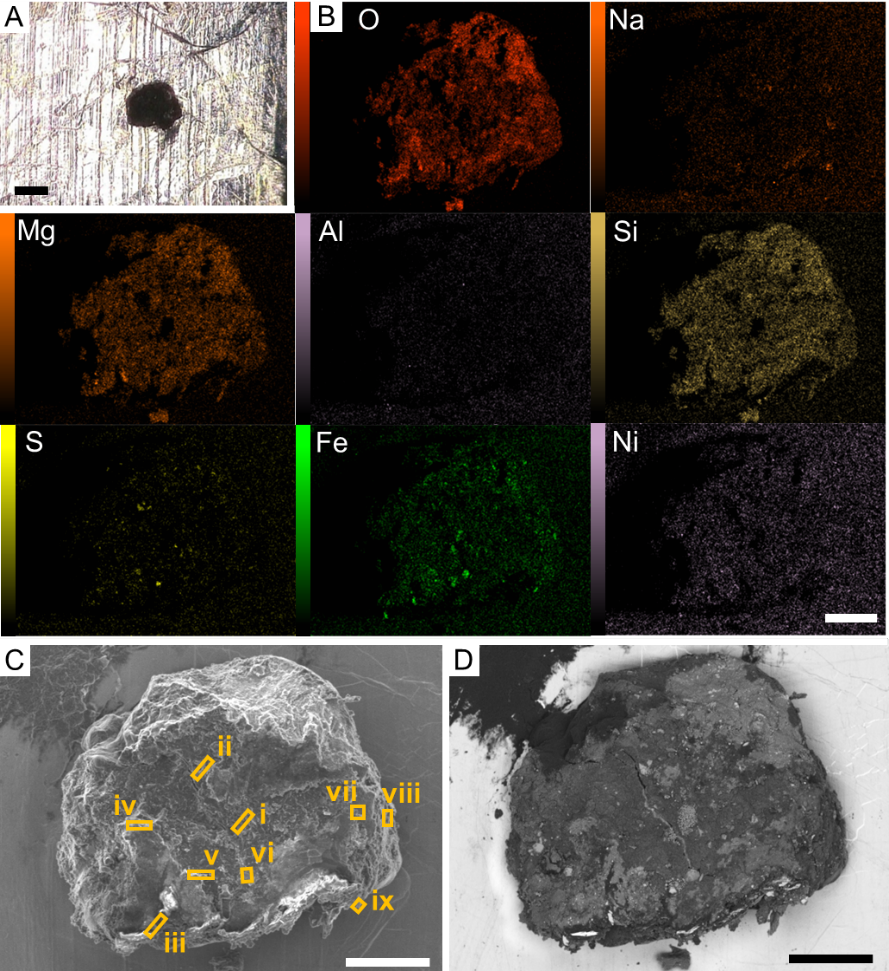


**Fig. S1. A piece (C0002-FC019) of the asteroid Ryugu.** (*A*) Optical microscopy image. (*B*) Elemental mapping by SEM-EDS analysis. (*C* and *D*) Secondary electron and back-scattered electron images, respectively. Thin sections were prepared from the rectangular regions i for magnetite (Fig. 1), ii for sulfide (Fig. 2), and iii for tiny iron–nickel sulfide particles (Fig. 3). Tungsten was deposited on the surface of candidate areas for FIB machining as indicated by the rectangular regions based on the abundances of S, Fe, and Ni. Scale bars are 100 μm for *A* and 40 μm for *B*–*D*. The corresponding atomic ratios are listed in Table S1.


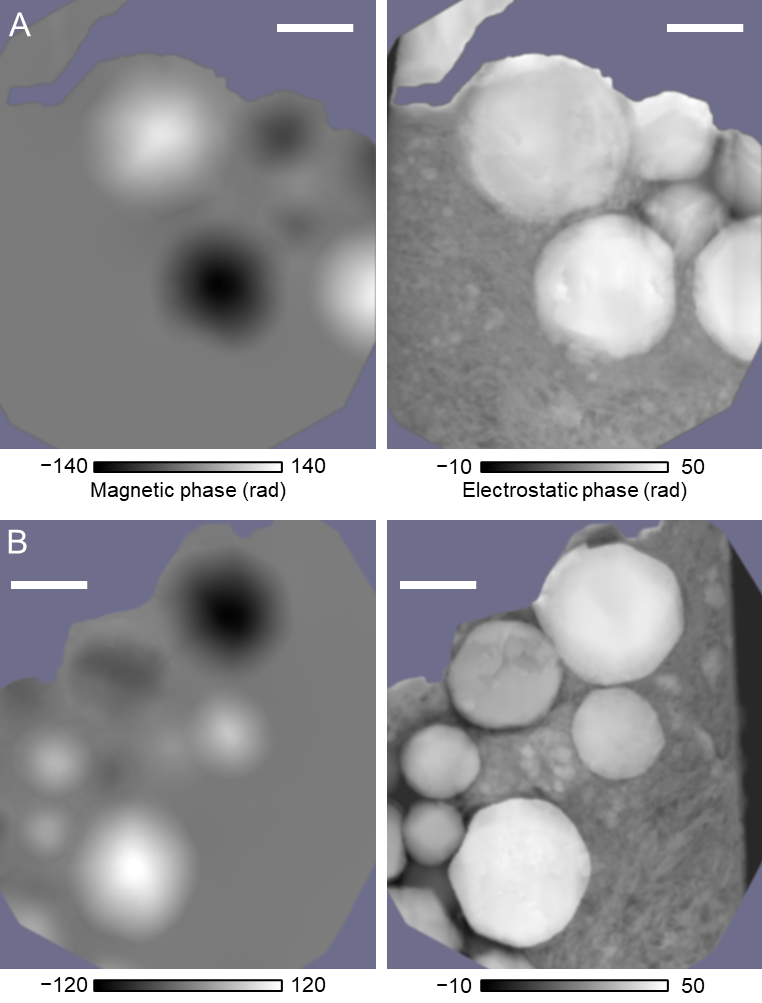


**Fig. S2. Magnetic (left) and electrostatic (right) phases of magnetites.** (*A* and *B*) corresponding to B and C in Fig. 1A. The bluish masked area indicates regions where hologram fringes were not detected. The scale bars are 500 nm.


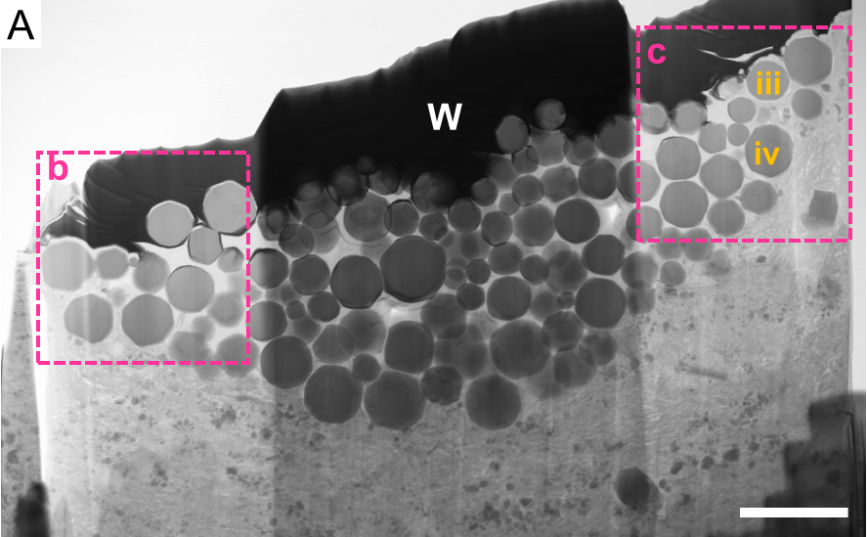

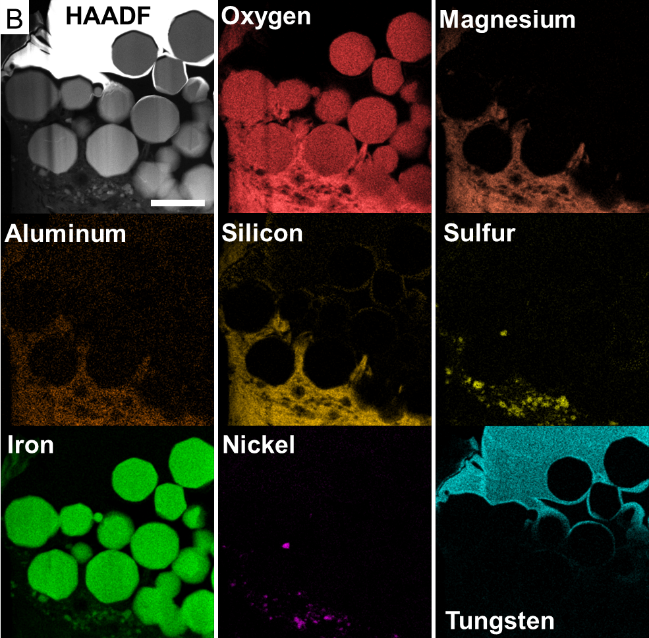

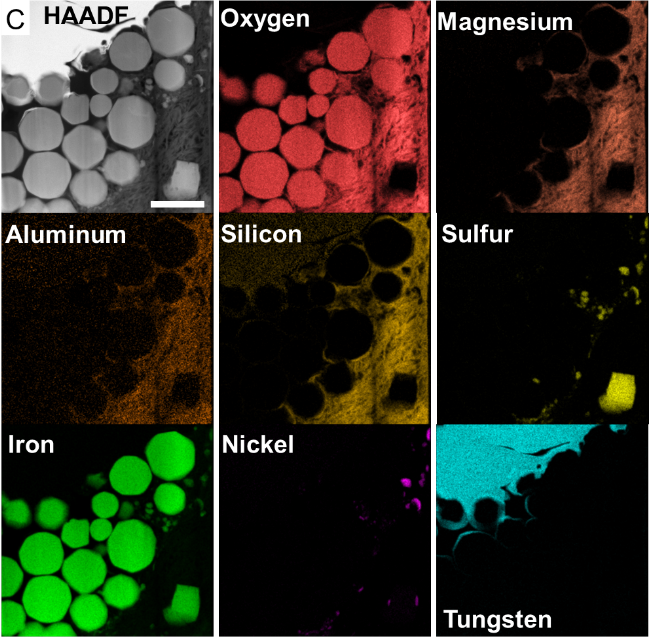


**Fig. S3. A thin section of a magnetite framboid observed by the JEM-F200 TEM.** (*A*) STEM bright-field image of the thin section in Fig. 1A after further thinning at both edges. The region with a strong contrast, W, is deposited tungsten layer to prevent beam damage during FIB machining. The scale bar is 2 μm. (*B* and *C*) Corresponding HAADF images and elemental mappings of the regions indicated by the dashed squares b and c, respectively, in *A*. Note that a 30-nm-thick layer of deposited carbon was present between the W layer and the original surface, and on the front and back faces. Corresponding TEM images and electron-diffraction patterns of iii and iv are shown in Fig. S4. The scale bars are 1 μm.


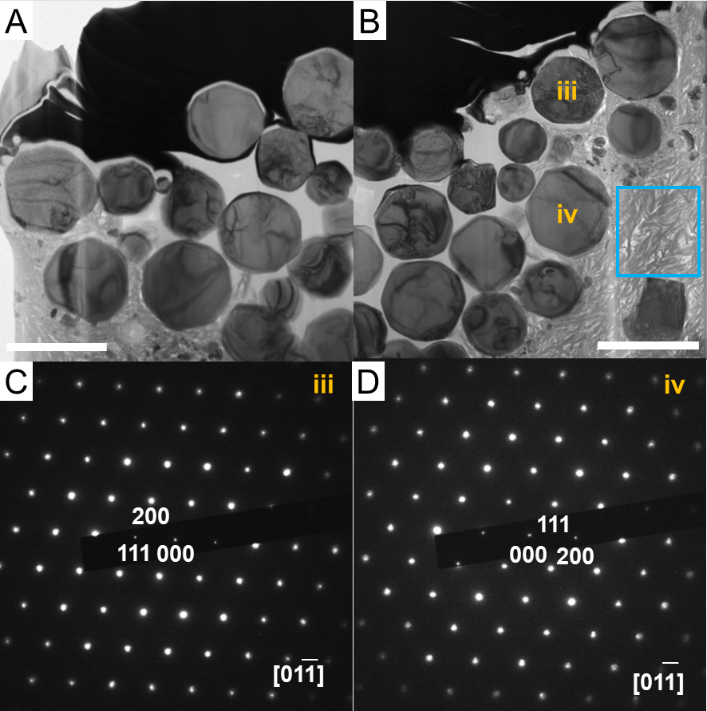


**Fig. S4. Enlarged TEM images of parts of a magnetite framboid, as observed by using the JEM-F200 TEM.** (*A* and *B*) TEM images corresponding to squares b and c, respectively, in the STEM images in Fig. S3*A*. The rectangle in *B* shows a typical phyllosilicate. Scale bars are 1 μm. (*C* and *D*) Electron diffraction patterns corresponding to iii and iv, respectively, in *B*.


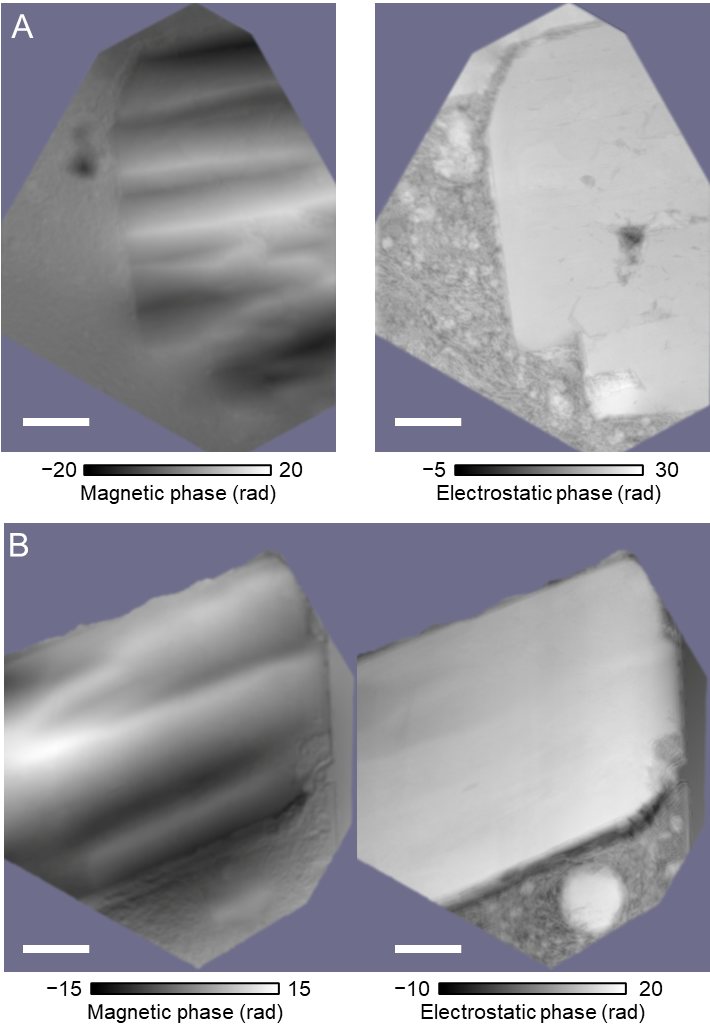


**Fig. S5. Magnetic (left) and electrostatic (right) phases of pyrrhotite.** (*A* and *B*) corresponding to b and c in Fig. 2A. The bluish masked area indicates areas where hologram fringes were not detected. Scale bars are 500 nm.


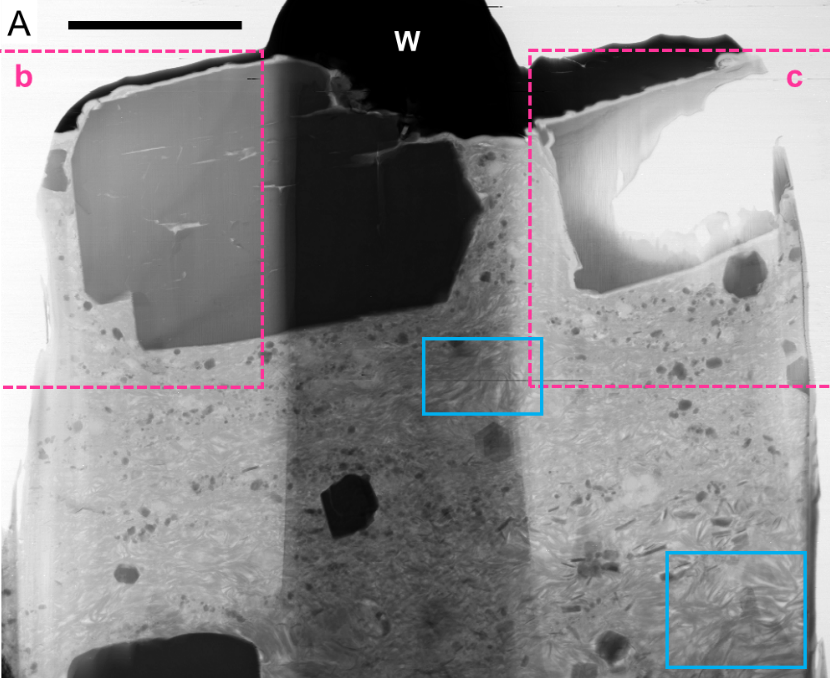


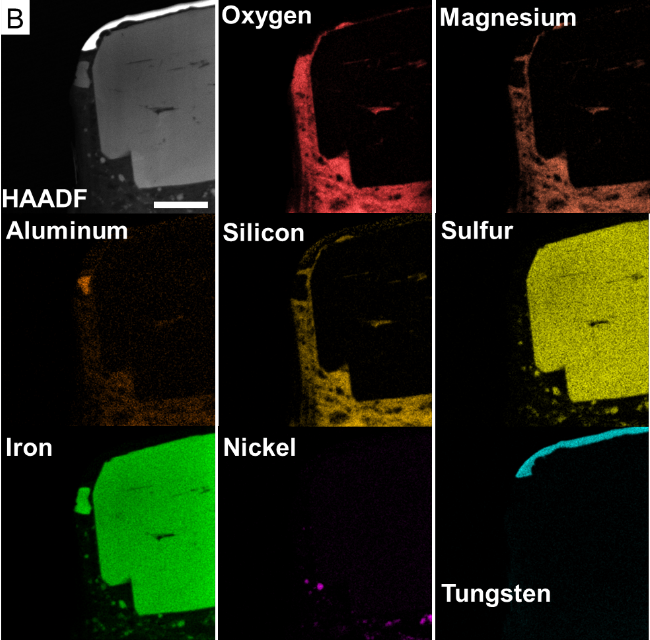

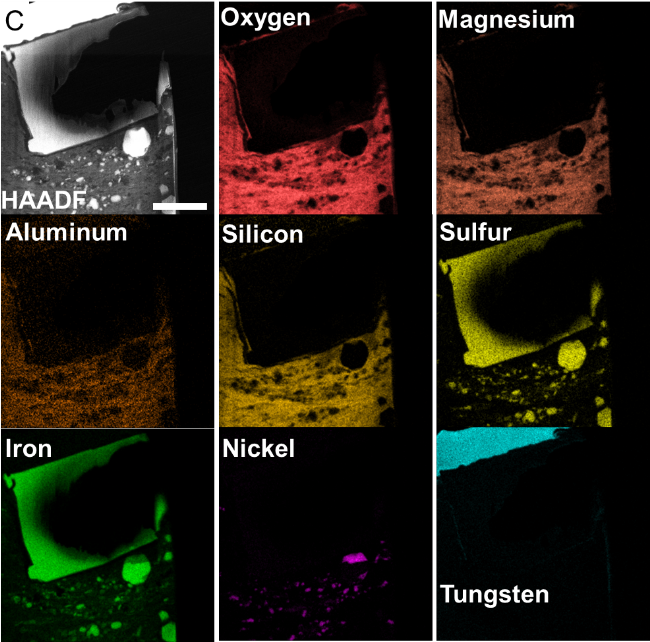


**Fig. S6. A thin section of sulfides after further thinning, as observed by using the JEM-F200 TEM.** (*A*) STEM-bright field image of the thin section in Fig. 2A after further thinning at both edges. The relatively strong contrast in the central region is due to a thick region retained to maintain the strength of the sample. The region with a strong contrast, W, is a layer of tungsten deposited to prevent beam damage during FIB machining. The solid rectangles (cyan) in A show structure of typical phyllosilicates. The scale bar is 2 μm. (*B* and *C*) Corresponding HAADF images and elemental mapping of the regions indicated by the dashed rectangles (pink) b and c, respectively, in *A*. The scale bars are 1 μm.


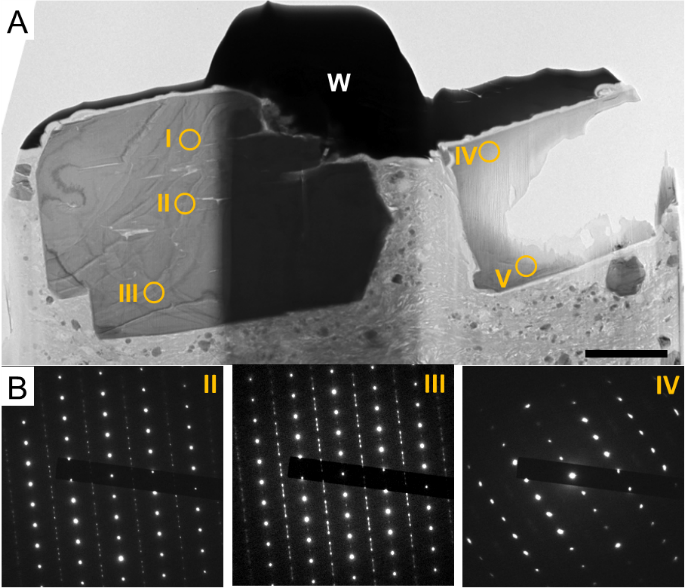


**Fig. S7. A thin section of sulfides after further thinning, as observed by using the JEM-F200 TEM.** (*A*) Bright-field TEM image of the thin section in Fig. 2A after further thinning at both edges. The relatively strong contrast in the central region is due to a thick region retained to maintain the strength of the sample. The region with a strong contrast, W, corresponds to a tungsten layer deposited to prevent beam damage during FIB machining. The scale bar is 1 μm. (*B*) Electron diffraction patterns corresponding to the regions II–IV in *A*. The diffraction spots correspond to polytype-4C, Fe_7_S_8_, of pyrrhotite. The sample was tilted to orient the crystal-zone axis parallel to the electron beam. The tilt angles (TX, TY) are (18.0, 15.5) for II, (21.5 and 12.6) for III, and (21.0, –24.0) for IV.


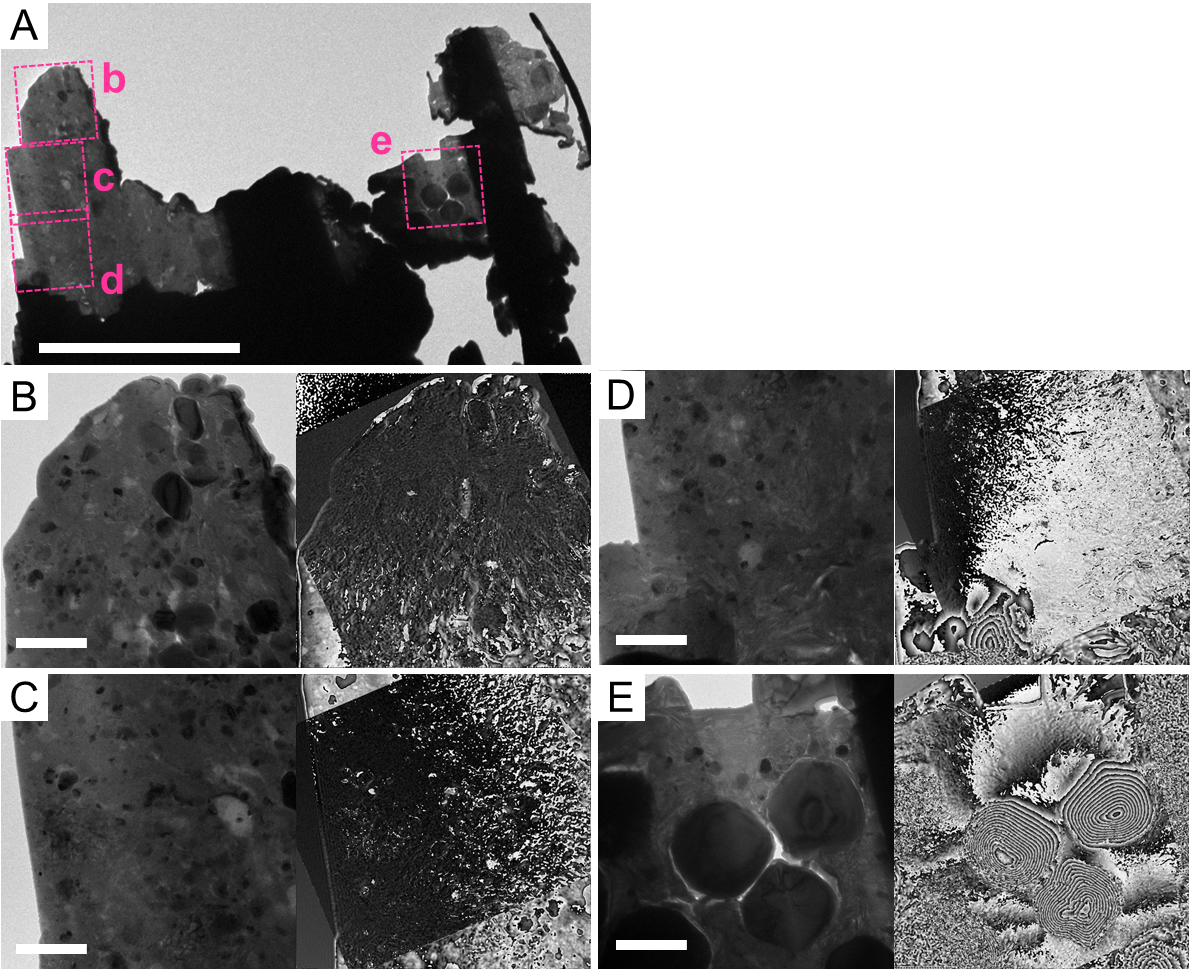


**Fig. S8. A thin section of tiny particles distributed in the matrix.** (*A*) TEM image of the whole of the thin section iii. The scale bar is 5 μm. (*B*–*E*) Magnified TEM images (left) of the square regions b–e in *A* and the corresponding magnetic-flux-distribution images (right) (two times the phase-amplified reconstruction). The bottom particles in *D* and the three particles in *E* show a concentric circular magnetic field and a closed (i.e., vortex) structure, characteristic of framboidal magnetite. The tiny particles do not show a significant magnetic field. The scale bars are 500 nm.


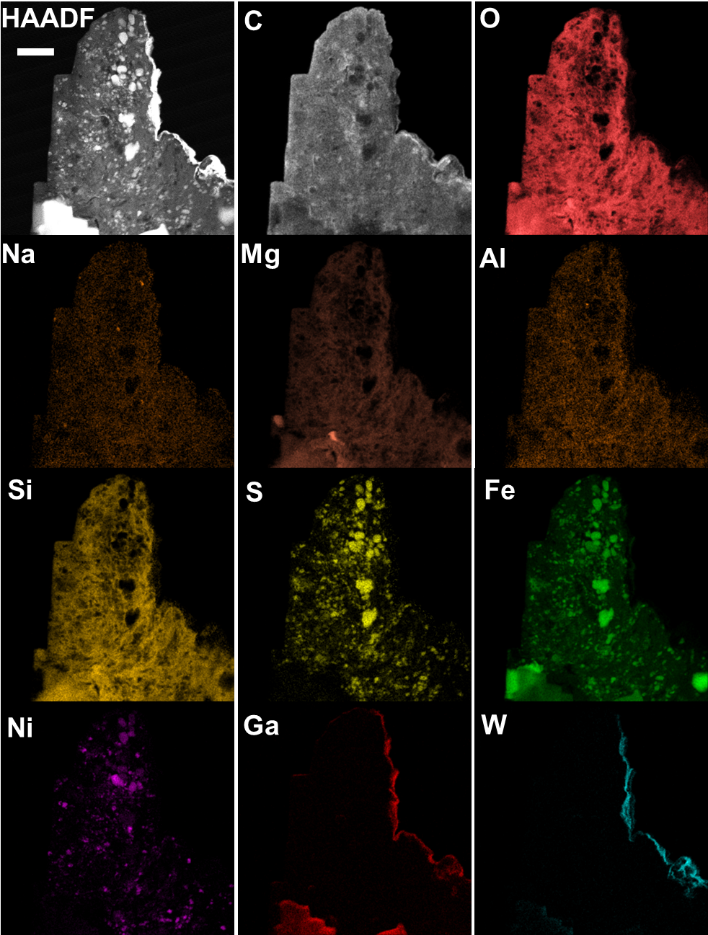


**Fig. S9. Analytical results of STEM-EDS for a thin section of the Fe-Ni-S region.** This section was observed by using the JEM-F200 TEM, corresponding to region iii in Fig. S1*C*. Tiny particles were distributed in the matrix. Corresponding images are presented in Fig. 3.

**Table S1.** Atomic ratios for the bulk C0002-FC019 sample and its relatively iron-rich regions, indicated as i–ix in Fig. S1.

| Element | Line | Bulk | i | ii | iii | Iv | v | vi | vii | viii | ix |  |
| --- | --- | --- | --- | --- | --- | --- | --- | --- | --- | --- | --- | --- |
| O | K | 53.4 | 51.0 | 17.2 | 26.7 | 51.7 | 29.5 | 52.5 | 39.1 | 51.0 | 20.3 |  |
| Na | K | 1.2 | 0.3 | n.d.^a^ | 1.1 | 0.9 | 0.6 | 0.6 | 1.3 | 0.3 | 5.1 |  |
| Mg | K | 14.4 | 5.2 | 7.7 | 3.6 | 16.1 | 8.4 | 4.1 | 10.7 | 5.5 | 3.4 |  |
| Al | K | 1.4 | 0.8 | 1.1 | 2.9 | 1.7 | 0.7 | 0.4 | 0.8 | 0.7 | 1.1 |  |
| Si | K | 15.8 | 5.7 | 10.1 | 7.2 | 16.0 | 7.2 | 5.5 | 11.2 | 5.6 | 7.4 |  |
| S | K | 4.0 | 0.7 | 43.6 | 1.4 | 4.4 | 25.9 | 0.2 | 13.7 | 1.1 | 4.7 |  |
| Fe | L | 9.0 | 35.0 | 19.9 | 50.2 | 8.2 | 26.8 | 36.4 | 21.7 | 35.5 | 47.3 |  |
| Ni | L | 0.8 | 1.3 | 0.5 | 7.0 | 1.0 | 1.0 | 0.4 | 1.5 | 0.4 | 10.6 |  |
| ^a^ Not detected. | | | | | | | | | | | | |

Table S2. Atomic ratios of each magnetite particle in Fig. 1.

| Element | Fe_3_O_4_ | I | II | III | IV | V | VI | Average | Error |
| --- | --- | --- | --- | --- | --- | --- | --- | --- | --- |
| O | 57.1 | 58.6 | 55.8 | 58.8 | 58.7 | 57.4 | 56.7 | 57.7 | 1.1 |
| Fe | 42.9 | 41.4 | 44.2 | 41.2 | 41.3 | 42.6 | 43.3 | 42.3 | 1.1 |
| Fe/O | 0.75 | 0.71 | 0.79 | 0.70 | 0.70 | 0.74 | 0.76 | 0.73 | 0.03 |

Table S3. Atomic ratios of iron sulfide particle in Fig. 2.

| Element | Fe_7_S_8_ | Fe_9_S_10_ | Fe_11_S_12_ | I | II | III | IV | V | VI | VII | VIII | IX |
| --- | --- | --- | --- | --- | --- | --- | --- | --- | --- | --- | --- | --- |
| S | 53.3 | 52.6 | 52.2 | 55.4 | 54.8 | 53.6 | 56.5 | 57.5 | 0 | 55.2 | 40.7 | 61.3 |
| Fe | 46.7 | 47.4 | 47.8 | 44.0 | 44.6 | 45.8 | 43 | 42 | 100 | 44 | 51.9 | 38.7 |
| Ni | – | – | – | 0.6 | 0.6 | 0.6 | 0.5 | 0.5 | 0 | 0.8 | 7.4 | 0 |
| Fe/S | 0.875 | 0.9 | 0.92 | 0.79 | 0.81 | 0.85 | 0.76 | 0.73 | – | 0.80 | 1.28 | 0.63 |

Table S4. Atomic ratios of the tiny particles in Fig. 3.

| Element | 1 | 2 | 3 | 4 | 5 | 6 | 7 | 8 | 9 | 10 |
| --- | --- | --- | --- | --- | --- | --- | --- | --- | --- | --- |
| S | 54.4 | 59.5 | 56.8 | 54.7 | 54.2 | 55.6 | 58.2 | 54.8 | 55.3 | 54.3 |
| Fe | 43.6 | 39.9 | 39.9 | 38.2 | 31.1 | 38.3 | 38.7 | 32.5 | 28.6 | 40.1 |
| Ni | 2.0 | 0.6 | 3.3 | 7.1 | 14.7 | 6.2 | 3.1 | 12.7 | 16.1 | 5.6 |
| Ni/(Fe + Ni) | 0.04 | 0.01 | 0.08 | 0.16 | 0.32 | 0.14 | 0.07 | 0.28 | 0.36 | 0.12 |

| Element | 11 | 12 | 13 | 14 | 15 | 16 | 17 | 18 | 19 | 20 |
| --- | --- | --- | --- | --- | --- | --- | --- | --- | --- | --- |
| S | 50.3 | 55.8 | 49.7 | 57.4 | 48.1 | 52.6 | 52.8 | 54.1 | 58.3 | 54.4 |
| Fe | 31.4 | 24.3 | 33.0 | 40.5 | 38.0 | 43.1 | 43.3 | 44.2 | 41.0 | 26.1 |
| Ni | 18.3 | 19.9 | 17.3 | 2.1 | 13.9 | 4.3 | 3.9 | 1.7 | 0.8 | 19.5 |
| Ni/(Fe + Ni) | 0.37 | 0.45 | 0.34 | 0.05 | 0.27 | 0.09 | 0.08 | 0.04 | 0.02 | 0.43 |
